# Lymphangiomatous endothelial cyst of the adrenal gland: A case report

**DOI:** 10.1016/j.eucr.2021.101859

**Published:** 2021-09-18

**Authors:** Rekik Issam, Mseddi Mohamed Amine, Triki Meriam, Chaabouni Ahmed, Bouassida Mehdi, Hadj Slimen Mourad

**Affiliations:** Habib Bourguiba Hospital, Urology Department, Sfax, Tunisia

**Keywords:** Adrenal gland, Adrenal cyst, Lymphangioma

## Abstract

Cystic adrenal tumors are rare with an incidence of approximately 0.06% in the general population. Four main histological types are distinguished: Endothelial cysts of lymphangiomatous or angiomatous origin, pseudocysts, epithelial cysts and cysts of parasitic origin. Surgery is recommended for signs of complications, suspicion of malignancy and large size. In other cases, simple surveillance can be proposed.

We report here a case of a lymphangiomatous endothelial cyst of the adrenal gland. The objective is to recall the clinical characteristics and to specify the diagnostic contribution of imaging as well as the therapeutic modalities of this entity.

## 1 Introduction

Cystic tumors of the adrenal gland are rare with an incidence of approximately 0.06% in the general population and may be discovered incidentally or be symptomatic.^1^ Classically, adrenal cysts have been classified into pseudocysts, endothelial cysts, epithelial cysts and parasitic cysts. Endothelial cysts are divided into lymphangiomatous and angiomatous.^2^

We report here a case of a lymphangiomatous endothelial cyst of the adrenal gland which aims to recall the clinical characteristics and to specify the diagnostic contribution of imaging as well as the therapeutic modalities of this entity.

## 2 Case presentation

This is a 46-year-old patient with no particular history who consults for right back pain. The clinical examination found a patient in good general condition, apyretic, with no palpable abdominal or lumbar mass and an unremarkable cardiovascular examination. Abdominal ultrasound showed the presence of a cystic tumor of the right adrenal gland measuring 35 mm in length.

**Fig. 1:**
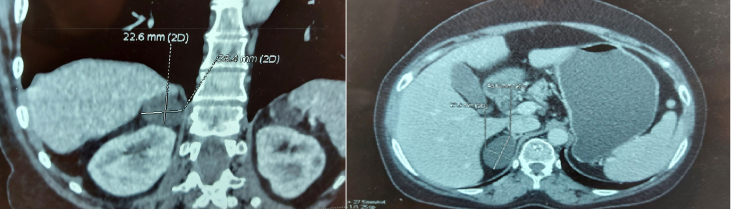
Computed tomography of a cystic right adrenal mass of oval shape and regular contours measuring 45*28*41mm.

In view of the persistence of low back pain, an abdominal tomography scan was performed, showing a cystic, oval mass of the right adrenal gland, with regular contours, 45 mm long axis, spontaneous density 10 HU with a relative washout of less than 50% as shown in Fig. 1.

Hormonal exploration was in favor of a non-secreting tumor, with aldosterone, renin, methoxylated derivatives, and dexamethasone suppression test all normal.

Given the increase in volume of the cystic mass, as well as the persistence of right lower back pain, the decision was to operate on the patient.

A right adrenalectomy was performed by laparoscopic transperitoneal approach, which was performed without any intraoperative incidents and with simple postoperative follow-up and resolution of the clinical symptoms.

**Fig. 2:**
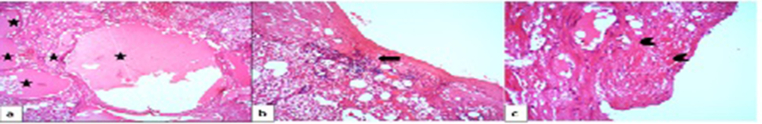
Pathological findings of cystic lymphangioma of the adrenal gland: a) multilocular cystic channels and spaces of varying sizes filled with eosinophilic, acellular lymph fluid (asterisk) (hematoxylin-eosin x50), b) the cystic wall is thick and contain small scattered lymphoid aggregates (arrow) (hematoxylin-eosin x100), c) note the presence of some smooth muscle fibers in the cystic wall (arrowhead) (hematoxyline-eosin x200).

Anatomopathological examination of the surgical specimen showed a lymphangiomatous endothelial cyst of the right adrenal gland without signs of malignancy, as illustrated in Fig. 2.

## 3 Discussion

Adrenal cysts are rare (0.064–0.18% of autopsy series).^3^ Only about 50 cases have been reported in the literature, but their incidental discovery is becoming more frequent with the advent of medical imaging techniques.

The sensitivity of their detection is 100% on MRI, 80% on computed tomography (CT) and 66.7% on ultrasound.^3^

The great majority of these cysts are small, unilateral and clinically asymptomatic. On the other hand, cysts larger than 10 cm in diameter may cause sensations of abdominal pain or tension, low back pain, or even gastrointestinal disorders due to compression of the proximal organs. The largest of these are sometimes accessible to abdominal palpation.^3^ More rarely, they may be the cause of an acute abdominal pain syndrome due to intracystic haemorrhage or of a state of shock due to retroperitoneal haemorrhage.^3^

In our case, the cystic mass was 45 mm in long axis on CT scan and the patient complained of right low back pain.

Histologically, four main types are distinguished^4^: Endothelial cysts (45%) of lymphangiomatous or angiomatous origin, pseudocysts, often highly vascularized and hemorrhagic (39%), epithelial cysts (9%) and cysts of parasitic origin, often hydatid (7%).

The anatomopathological examination of the surgical specimen of our case concluded to a lymphangiomatous endothelial cyst without signs of malignancy.

Radiological studies can be useful for the pre-surgical diagnosis of cystic lesions of the adrenal gland. Computed tomography (CT) has been shown to be very effective in recognizing cystic lesions, but current imaging modalities cannot clearly distinguish benign from malignant adrenal cysts.^5^

The histologic nature of the cystic adrenal lesion is often difficult to specify with simple imaging. Only the presence of parietal calcifications suggests an adrenal pseudocyst.^5^

In our case, the CT scan confirmed the cystic nature of the adrenal mass, which did not present signs of malignancy, but could not predict its histological nature.

Careful analysis, both morphological and hormonal, is necessary in the face of any incidental finding of a cystic adrenal image. The diagnosis of pheochromocytoma necessarily leads to surgical removal of the tumor. In the absence of pheochromocytoma, surgery is recommended (unilateral adrenalectomy) in the presence of signs of complications, suspicion of malignancy and large size (greater than 5 cm). In other cases, simple clinical and CT surveillance can be proposed.^4^

In our patient, we opted for a surgical treatment consisting in a right adrenalectomy by laparoscopic transperitoneal approach, in view of the increase in size of the cystic mass and the persistence of low back pain in our patient. The evolution was favorable, with simple postoperative courses, and without signs of recurrence over a 5-year follow-up.

## 4 Conclusion

Cystic adrenal lesions are rare, covering a broad spectrum of primary adrenal tumors and may be associated with both benign and malignant adrenal tumors.

Distinction between adrenal cysts and adrenal tumors with cystic changes should be made by extensive pathologic sampling of any macroscopically suspicious lesion.^4^

Surgery is recommended if there is evidence of complications, suspicion of malignancy, and large size (greater than 5 cm). In other cases, simple clinical and CT surveillance can be proposed.^3^
